# Exploring the impact of various cooking techniques on the physicochemical and quality characteristics of camel meat product

**DOI:** 10.5713/ab.22.0238

**Published:** 2023-06-26

**Authors:** Mouza Bahwan, Waqas N Baba, Oladipupo Adiamo, Hassan Mohammed Hassan, Ume Roobab, Olalere Olusegun Abayomi, Sajid Maqsood

**Affiliations:** 1 Department of Food Science, College of Agriculture and Veterinary Medicine, United Arab Emirates University, Al Ain, 15551, United Arab Emirates; 2 Centre for Nutrition and Food Sciences, Queensland Alliance for Agriculture and Food Innovation (QAAFI), The University of Queensland, Coopers Plains, Brisbane, QLD 4108, Australia; 3 Analytical Biochemistry Research Centre (ABrC), Universiti Sains Malaysia, University Innovation Incubator Building, Sains@USM, 11900 Bayan Lepas, Penang, Malaysia

**Keywords:** Biochemical Changes, Camel Meat Product, Cooking Methods, Lipid, Protein, Textural Characteristics

## Abstract

**Objective:**

The objective of this study was to evaluate the effects of four different cooking techniques viz: boiling, grilling, microwave, and frying; on the physicochemical characteristics of camel meat.

**Methods:**

Protein composition and their degradation as well as biochemical and textural changes of camel meat as influenced by cooking methods were investigated.

**Results:**

The highest cooking loss (52.61%) was reported in microwaved samples while grilled samples showed the lowest cooking loss (44.98%). The microwaved samples showed the highest levels of lipid oxidation as measured by thiobarbituric acid reactive substances, while boiled samples showed the lowest levels (4.5 mg/kg). Protein solubility, total collagen, and soluble collagen content were highest in boiled samples. Boiled camel meat had lower hardness values compared to the other treated samples. Consequently, boiling was the more suitable cooking technique for producing camel meat with a reduced hardness value and lower lipid oxidation level.

**Conclusion:**

The camel meat industry and camel meat consumer can benefit from this research by improving their commercial viability and making consumers aware about the effects of cooking procedures on the quality of camel meat. The results of this study will be of significance to researchers and readers who are working on the processing and quality of camel meat.

## INTRODUCTION

Meat is an excellent source of nutrients, particularly high-quality protein, which is abundant in essential amino acids and has a higher bioavailability than other protein sources. It contains a high saturated fatty acid (SFA) content, which is regarded as a risk factor for chronic illnesses to consumers. Consuming meat from sources with a lower SFA content, which is considered a healthier choice, is an alternate technique for avoiding the danger associated with SFA. It is important to know that camel meat provides lesser fat and cholesterol and more polyunsaturated fatty acids (PUFA) than that of other commercial red meats [1]. Furthermore, new meat sources are being continuously explored as rich protein sources to meet the increasing protein demand [2]. However, newly explored meat sources such as rabbit [2] and cane rat [3] have limited prospects as a sustainable food source due to their smaller body size in comparison to commonly used farm animals, unlike the prospect of camels. Consumption of camel meat is popular in many Asian and African countries due to leanness. However, camel meat is perceived to be tough, fibrous, and having a peculiar off-odor. According to Maqsood [1], such off-odors may result from insufficient preservation and storage of camel meat, and its high susceptibility to lipid oxidation.

Furthermore, cooking causes changes in the physicochemical, biochemical, and sensory properties of meat, which improves its hygienic quality, flavour, taste, and shelf life [4]. Cell membrane disintegration, collagen solubilization, protein gelation, and the synthesis of flavours all contribute to the ideal organoleptic qualities present in the finished product. Cooking, on the other hand, may cause certain undesired modifications in meat proteins and lipids that vary across cooking techniques and may have an unfavourable impact on meat quality. Moreover, protein oxidation, degradation, denaturation, and aggregation may all be induced by different cooking regimens. Also, lipid oxidation of pork cooked using different cooking treatments adversely affects the fatty acid profile and shelf life of pork differently [5]. Meat high in unsaturated fatty acid content such as rabbit meat were adversely affected by generation of free radicals induced by cooking and had a detrimental effect on the shelf life [4]. Such changes may produce a nutritionally inferior end-product under intensive cooking conditions. The effects of various cooking methods such as grilling, roasting, and frying on various biochemical properties of goat [6], beef [7], foal meat [8], rabbit meat [2], seafood [9], lamb [10], and chicken meat [11] have been reported [12].

Camel meat is gaining considerable importance due to its lower fat content compared to other meat types such as beef and sheep. Moreover, camel meat has lesser cholesterol and high levels of PUFA, making camel meat superior in terms of health benefits [1]. However camel meat is generally presumed to be tougher than the other commonly consumed meat types [13]. There are some preliminary studies that suggest pretreatments and cooking methods affect various quality attributes of camel meat. Treatments such as roasting, and microwave were reported to cause significant structural damage and fiber shrinkage in the meat. Similarly, Kenenbay et al [14] investigated the technological properties of camel meat, determining the amount of meat losses and the duration of heat treatment during boiling and roasting. According to the authors, camel meat loses 35.95% of weight during roasting, while much of its fat goes into the broth. A more significant loss of protein occurs during boiling (7.6% to 9.8%) compared to roasting (5.0% to 6.0%) owing to the transition of soluble proteins into broth. In another study salting and smoking was reported to affect the acceptability of camel meat [15]. Despite the higher PUFA that are prone to lipid oxidation, the effect of various cooking methods on the extent of lipid oxidation have not been reported. In addition, there is no information on the changes in protein profile of camel meat as influenced by commonly used cooking methods. Evidently, a detailed account of various cooking methods on physicochemical and quality attributes is lacking and due to which a firm conclusion about the most suitable method for cooking camel meat is obscure.

The current research aimed to assess the impact of different cooking techniques, including grilling, frying, microwave heating, and boiling on development of lipid oxidation and as well as modifications in protein and textural characteristics in camel meat. This research revealed important procedures to address the usual limitations (toughness and off-odor) that impair consumer preferences for camel meat.

## MATERIALS AND METHODS

### Chemical and reagents

The chemicals used in this study were either from Sigma Aldrich (St. Louis, MO, USA) or BDH (Middle East, Dubai, UAE). All the reagents for electrophoresis were purchased from Bio-Rad (Richmond, CA, USA).

### Preparation of samples

Five female camels (age 3 to 5 years) were slaughtered at a local slaughterhouse in Al Ain, UAE following the UAE-Standard No. 993/2000 as per Islamic law. Five camels used in this study were considered to be fairly sufficient as this study mainly deals with the effect of processing conditions (cooking) on the biochemical properties. This particular age group was selected on the basis of previous studies that have included similar age group (3 to 5 years) [15]. The carcasses were stored for 24 h at 2°C. The Adductor muscles from both sides (n = 10) were dissected from inside round muscles and were packed in a polyethylene bag and transported chilled to the laboratory of the Food Science Department. The muscles were cut into similar sized cubes (2×2×2 cm^3^). The meat cubes were randomly assigned into fifteen groups that were randomly assigned to five treatments representing four cooking methods and fresh meat samples that served as a control with 3 replicate samples with each treatment. Heat treatment was considered complete when all the samples had reached an internal temperature of 70°C to 75°C [4], which was monitored by a thermocouple thermometer. Grilling was carried out at 180°C to 200°C for 7.5 min on each surface of the meat cube samples using an electrical griddle. For microwaved meat samples, each surface of the sample was microwaved (Miele Contour Line M6012; Miele, Gütersloh, Germany) at 900 W for 1.5 min. Frying was done using 15 mL refined olive oil purchased from a local supermarket in Al –Ain (UAE), at a temperature range of 170°C to 180°C for 6 min on each surface using a frying pan (Prestige, Bangalore, India) with the dimensions 6.4×42.6×28.6 cm. Boiling was carried out for 30 min using a pressure cooker (PR100-6, Quart; Black & Decker, OH, USA) containing 2 L of water. Cooked samples were immediately studied for cooking loss and textural analysis. For the rest of the analysis, samples were cooled, packed into zipped HDPE polyethylene bags (9″×12″) and stored at −20°C for further analysis.

### Proximate composition

Proximate composition of uncooked and cooked meat samples was analyzed following AOAC procedures [16]. The sample was analyzed for total moisture content at 110°C for 24 h (AOAC, 950.46B). The total protein content of samples was determined by the Kjeldahl method (AOAC, 928.08). Total lipid content was determined by the Soxhlet method (AOAC, 991.36) and the total ash content was determined as per AOAC, 920.153.

### Cooking loss

Cooked samples were cooled at room temperature for 20 min and the cooking loss was calculated as the percentage weight difference between fresh and cooked samples relative to the weight of fresh meat samples as follows:


$$Cooking\;loss=\frac{Raw\;meat\;weight-cooked\;meat\;weight}{Raw\;meat\;weight}\times 100$$

### Total protein solubility

Total protein solubility was determined by the method described by Joo et al [17]. Total protein solubility was measured as mg of protein/g of sample.

### Total collagen content and hydroxyproline determination

Meat samples were analyzed for hydroxyproline (HP) content according to the procedure suggested by Naveena and Mendiratta [18]. The standard solution consisted of HP with concentrations ranging from 10 to 60 ppm. Total collagen (mg/g wet sample) was calculated as:


Total collagen=HP×8.0

### Collagen solubility

Collagen solubility was determined by analyzing soluble HP content in the supernatant and was calculated as per the method of Williams and Harrison [19]. The collagen solubility was expressed as the percent (%) soluble collagen of the total collagen present in the fresh camel meat.

### Sodium dodecyl sulfate polyacrylamide gel electrophoresis

Cooked and uncooked meat samples were subjected to sodium dodecyl sulfate polyacrylamide gel electrophoresis (SDS–PAGE) according to the method described by Maqsood and Benjakul [20]. Briefly, about 3 g of sample mixed with 27 mL of 5% SDS solution was homogenized (13,500 rpm, 2 min). The homogenate was incubated (85°C) for 1 h followed by centrifugation (3,500 g, 20 min). Sample (15 μg) was loaded on to a polyacrylamide gel and subjected to electrophoresis at 15 mA current using a Mini Protein II unit (BioRad, Richmond, CA, USA). The separated proteins were stained with 0.02% coomassie brilliant blue. A wide range of molecular weight markers were used for the estimation of the molecular weight of proteins.

### Thiobarbituric acid reactive substances

Camel meat samples were analyzed for thiobarbituric acid reactive substances (TBARS) following the method described by Maqsood and Benjakul [21]. A standard curve was prepared using 1,1,3,3-tetramethoxypropane with concentration ranging from 0 to 10 ppm and TBARS was expressed as mg of malonaldehyde (MDA) equivalents/kg sample.

### Texture profile analysis

Texture profile analysis (TPA) of camel meat samples was studied using a texture analyzer (Brookfield CT3 Texture Analyzer; CT3-4500, Brookfield Engineering Laboratories, Middleboro, MA, USA) as described by Maqsood et al [22]. Cooked and raw camel meat samples were tested using a cylindrical aluminum probe (TA4/1,000). The tests were performed with two compression cycles at room temperature with the following testing conditions: pretest, test, and return-test speed of 2.0 mm/s, a target distance of 7.0 mm, trigger load of 4.0 g, and the time interval between the first and the second cycle was 1 s. Texture Expert version 1.0 software (Stable Micro Systems, Surrey, England) was used to collect and process the data. Hardness (maximum force required to the first compress the samples in g), cohesiveness (the ratio of positive force area during the second to that the fist compression cycle), springiness (ratio of the time duration of force input during the second to that during the first compression), and chewiness (hardness×springiness×cohesiveness; multiply hardness, springiness and cohesiveness) of each sample was calculated from respective force-time curves generated.

### Statistical analysis

All the experiments were conducted in triplicate using three different batches (replicates) for each treatment (cooking methods and fresh camel meat). Experimental data was analyzed by the general linear model using one-way analysis of variance. The data was subject to post-hoc analysis using Duncan’s test. Differences were considered significant if p<0.05. All the data analysis was carried out using the IBM SPSS Statistics 22 software package (IBM Corp, Armonk, NY, USA; 2016).

## RESULTS AND DISCUSSION

### Effect of cooking methods on proximate composition of camel meat

The proximate composition of fresh and cooked meat samples is presented in Table 1. Proximate composition of the raw camel meat was found to be similar to that reported previously for dromedary camel meat [23], which documents the low lipid content in camel meat. As expected, cooking decreased the moisture content while protein, ash and fat contents were increased (p<0.05). Among the cooked samples, boiled and fried samples showed the highest and lowest moisture contents, respectively (p<0.05). Juárez et al [24] also reported higher moisture loss in fried buffalo meat while boiled samples showed the lowest moisture loss. During boiling, the samples are immersed in water, and meat principally consists of proteins with tendency to absorb water resulting in high moisture content of boiled samples. Frying does not involve water and as such application of heat results in drying of samples resulting in lower moisture content in comparison to boiled samples. The decrease in the moisture content in cooked meat samples is considered as the most prominent change that affects the overall proximate composition and subsequently the nutritional value of the consumed meat. It has been reported that during cooking, thermal denaturation of meat proteins leads to entrapment of a lesser amount of water than uncooked meat proteins with concurrent shrinkage that lead to translocation of the moisture from the muscle lattice to the cooking medium [24]. The fat content of the fried meat samples was significantly (p<0.05) higher than the rest of the other treatments. The increase in the fat content in fried camel meat is due to absorption of oil used during the frying process. Furthermore, frying involves loss of water by evaporation, which increases oil penetration into the food resulting in an increase in the total fat content. Boiled samples showed the lowest total ash content (p<0.05) that can be due to leaching of minerals during cooking in boiling water under high pressure.

### Influence of cooking methods on cooking loss of camel meat

Cooking loss refers to the loss of liquid and soluble matter from a meat sample during cooking, which results in decreased water content and hence a proportional increase in the fat and protein contents. During cooking, water is the main component that is lost. Cooking loss in camel meat samples due to different cooking methods is presented in Table 1. Different cooking methods display variable cooking losses that might be due to different degrees of heat-induced protein denaturation which causes a varied amount of water to be entrapped within the meat protein structures [24]. Mass transfer during cooking also plays an important role in determining the cooking loss and therefore different cooking methods lead to different cooking losses. Microwaved samples significantly show the highest cooking loss (52.61%), followed by boiled (47.10%), fried (47.66%) and grilled samples (44.98%). However, there is no significant (p>0.05) difference was found in cooking loss of fried, grilled, and boiled samples. This effect in adductor muscle is contradictory to that reported in camel *Longissimus dorsi* (LD) muscle where microwaved samples compared to braised and roasted samples [25]. Domínguez et al [4] also reported significantly (p<0.05) higher cooking loss in microwaved foal samples than grilled (22.45%±5.51%) and fried ones (23.73%±2.87%). Similar results were also reported in microwaved beef and pork steaks [5]. Sánchez-Muniz and Bastida [26] suggested that higher cooking loss in microwaved meat samples may be due to the absence of crust formation unlike grilling and frying where a crust is formed that slows down the exit of seeping liquid [5]. Furthermore, during microwave cooking, it is likely that a short time, high power, and electromagnetic field causes rapid protein denaturation and degradation that results in higher losses of aqueous and non-aqueous matter. Grilling and roasting require a longer time to achieve the final core temperature, therefore, the proteins do not undergo denaturation rapidly, resulting in the lower cooking loss. Boiling involves cooking meat samples in boiling water under pressure, which retards the loss of water from the samples during cooking, as the samples always remain surrounded by water throughout the cooking process. Also, the time taken to reach the core temperature of 70°C is higher in the case of boiling than microwave cooking. As such, protein denaturation occurs slowly during boiling that further decreases in water loss from the meat samples.

### Effect of cooking technique on protein solubility of camel meat

**Figure 1:**
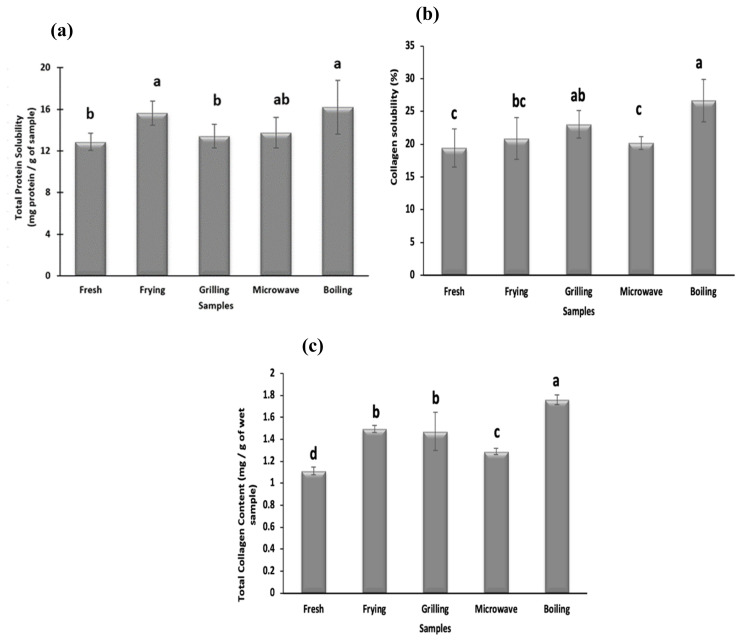
Effect of cooking techniques on (a) total protein solubility, (b) total collagen content, and (c) collagen solubility in camel meat. ^a–d^ Values with different letters are significantly (p<0.05) different from each other.

**Figure 2:**
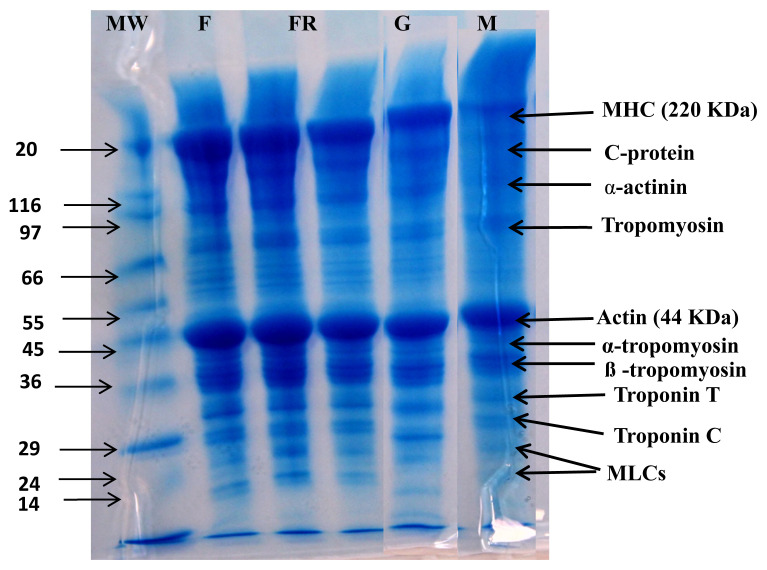
Effect of cooking techniques on Changes in proteins as depicted by sodium dodecyl polyacrylamide gel electrophoresis (SDS-PAGE) in camel meat subjected to different cooking techniques. MW, molecular weight of protein marker; F, fresh camel meat; FR, fried camel meat; G, grilled camel meat; M, microwaved camel meat; B, boiled camel meat.

Protein solubility is an important physicochemical property, which is a function of myofibrillar protein degradation [1]. As shown in Figure 1a all cooking methods significantly increase the protein solubility of meat samples. The highest protein solubility is seen in boiled samples while grilled and microwaved samples show the lowest values (p<0.05). An increase in the protein solubility in camel meat samples during cooking has been previously reported. Different cooking methods may disrupt the structure of meat proteins that increase protein solubility values. Protein solubility values obtained in cooked meat samples are in affirmation to the protein patterns observed in SDS-PAGE that show the highest protein degradation in the boiled samples (Figure 2).

### Effect of cooking methods on collagen content and collagen solubility

Collagen content plays an important role in meat tenderness and texture. The total collagen content of raw and cooked camel meat samples is shown in Figure 1b. The value of total collagen content in fresh camel meat (1.12 mg/g of wet sample) which is comparable to collagen content (1.6 to 2.3 mg/g of wet sample) reported in LD muscle of camel and cattle (1.63 to 1.86 mg/g) by Eskandari et al [27]. However, the total collagen content present in the camel meat was lower than chicken, lamb and buffalo [28]. The collagen contents of cooked camel meat samples were significantly (p<0.05) higher than raw meat samples. The highest value of total collagen content is reported for boiled samples (1.76 mg/g) which is significantly (p<0.05) higher than fried (1.49 mg/g), microwaved (1.28 mg/g) and grilled (1.42 mg/g) samples. The increase in total collagen content with increase in the temperature during water bath heating up to 80°C and microwave heating was also reported in beef semitendinosus muscle [29]. This increase was attributed to the higher cooking loss and the conversion of collagen to gelatin during heating. Cooking may lead to deterioration of intermolecular linkages of collagen fibrils that enables increased extraction of collagen levels, reflected as an increased concentration of HP during the analysis. It may also be mentioned that leaching of collagen along with the cooking loss was not reported to affect the total collagen values during the conventional heating method [27].

The degree of collagen solubility is largely used for the assessment of meat tenderness. All cooking methods increase the soluble collagen content of meat samples (Figure 1c), except frying and microwaving that do not vary significantly from fresh samples. Boiled samples show the highest collagen solubility (26.6%) compared to fried (20.8%), grilled (23.02%) and microwaved (20.14%) samples which can be attributed to longer cooking time under high pressure involved during boiling. Vasanthi et al [28] also reported the highest collagen solubility at 100°C and suggested maximum conversion of collagen to gelatin at this temperature. The soluble collagen content of pork was also reported to increase with the increase in heating time and temperature [29]. Kong et al [30] also reported maximum collagen solubilization in chicken and salmon after 20 min of cooking. Nikmaram et al [25] also reported higher collagen solubilization during braising and roasting than microwave cooking of camel and veal, which further supports the findings of this study. Therefore, cooking treatment, especially boiling caused collagen to degrade resulting in an increase in solubility of collagen.

### Influence of cooking methods on lipid oxidation of camel meat

**Figure 3:**
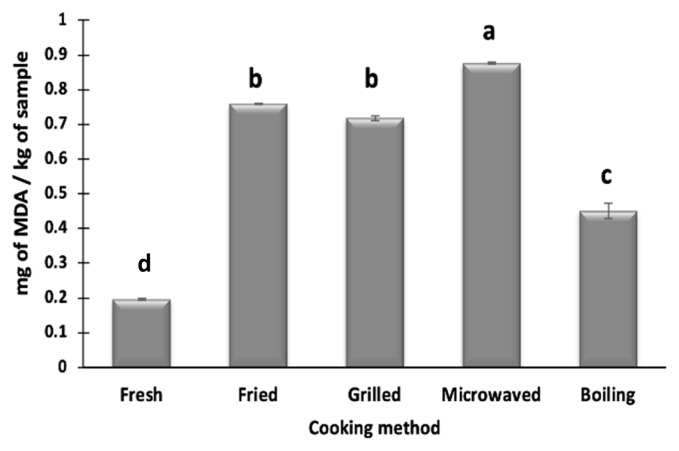
Effect of cooking techniques on thiobarbituric acid reactive substances (TBARS) in camel meat. ^a–c^ Values with different letters are significantly (p<0.05) different from each other.

Lipid oxidation in camel meat was measured in terms of mg of MDA/kg of sample using TBARS index. Djenane et al [13] also used TBARS to measure the lipid rancidity in camel meat and associated it to the high PUFA levels of camel meat. PUFA due to high level of unsaturation are highly sensitive to oxidation that can adversely affect the product shelf-life. All the cooking methods resulted in a significant increase in MDA formation of camel meat (Figure 3), which can be due to an increase in oxidation of meat at high temperatures. Similar trend was reported previously in foal meat [16] and silver catfish fillets [31] that were cooked using different methods. Higher levels of TBARS indicate the formation of secondary lipid oxidation products [21] which contributes to the off-odor development in the meat as well as health risks. MDA formation in fresh and cooked camel meat is higher than the values reported previously for lamb, goat, buffalo, foal meat, which may be attributed to the high levels of PUFA in camel meat. Moreover, camel meat contains high amounts of myoglobin and other haem compounds that act as prooxidants, thus it promotes lipid oxidation [1]. Among the different cooking methods used, the highest MDA formation is observed in microwaved samples (0.87 mg/kg), while the lowest in boiled samples (0.45 mg/kg). The formation of high MDA levels during the microwave treatment has been previously reported in foal meat and silver catfish fillets [23]. Domínguez et al [4] suggested higher MDA formation in microwaved meat samples may be due to the interaction of electromagnetic waves and fat that lead to oxidation of PUFA. A non-significant (p>0.05) difference in MDA formation between fried (0.76 mg/kg) and grilled (0.71 mg/kg) samples is seen but these values are significantly (p<0.05) lower than microwave-treated samples. Grilling involves longer treatment time (7.5 min) than microwaving, during which the oxidation products may react with amino-acids or other molecules resulting in a decreased MDA content. Weber et al [31] attributed lower formation of MDA in fried samples than microwaved samples to the dissolution of MDA into frying oil. Thus, depending upon the cooking method, cooked camel meat samples show different degrees of lipid oxidation that is below the animal product standards (1.5 to 2.0 mg/kg) among which microwaved samples had higher lipid oxidation.

### Protein pattern as detected by sodium dodecyl sulphate-polyacrylamide gel electrophoresis

The effect of different cooking methods on protein degradation of camel meat is depicted in Figure 2. The detectable protein bands in fresh camel meat included myosin heavy chain (MHC) (220 kDa), C-protein, α-actinin, tropomyosin, actin (44 kDa), α-tropomyosin, β-tropomyosin, Troponin T and C, and myosin light chains that are in conformity to our previous reports on camel meat [1]. Cooking causes varying changes in detectable protein bands of camel meat samples depending on the cooking method used. A degradation of proteins in meat with cooking was previously reported in beef [32]. Microwaving and boiling resulted in noticeable protein degradation compared to fried and grilled samples. Boiling produces prominent degradation in MHC, C-protein, tropomyosin, α-actinin, and considerable degradation is also seen in Troponin C, α-tropomyosin, β-tropomyosin, and troponin T. However, actin due to its high thermal stability is slightly affected. The noticeable degradation of proteins in boiled camel meat may be due to exposure of camel meat to high temperature and pressure for a longer time (30 mins) compared to the other cooking methods. Murphy and Marks [33] also reported an increase in protein degradation of ground chicken patties at boiling temperature (80°C). Zhang et al [34] also reported an increase in degradation of rabbit meat proteins with an increase in treatment time, however, they reported greater degradation in fried samples, which can be due to differences in cooking methodology (wet vs dry) and samples used. The higher protein degradation in boiled camel meat correlates well with the lower hardness values in the TPA (Table 2). Therefore, boiling was able to degrade the protein that positively affects the tenderness of camel meat.

### Influence of cooking methods on the textural profile of camel meat

The effect of cooking methods on the textural properties of camel meat was measured in terms of hardness, cohesiveness, springiness, and chewiness (Table 2). Cooking significantly increases all the textural attributes of camel meat. Meat’s texture was closely associated with the properties of muscle protein, and the inner connective tissue network. Thermal treatments induce myofibril contraction and collagen denaturation that alters the textural attributes of meat. Tougher texture suggests lesser water holding capacity and as such greater cooking loss that results in less tender cooked samples [6].

Boiled samples show lower hardness values (1,923.5 g) than fried (2,327.3 g), grilled (2,122.4 g) and microwaved (2,716.5 g) samples (p<0.05). Among the cooked samples, boiled samples display lowest springiness (7.80 mm), cohesiveness (0.76) and chewiness (70.13 mJ) values compared to other cooking methods (p<0.05). Yarmand and Homayouni [35] reported that microwave treatment separated fat cells from the muscle matrix and enhanced cooking loss, which in turn increased the hardness values. Among different cooking methods, boiling involves high pressure, a longer time and lower temperature (i.e., slow cooking under high pressure) that positively affects the textural parameters. Slow cooking has been related to collagen solubilization that mostly happens in the temperature range of 60°C to 80°C with long exposure time [36]. According to Vasanthi et al [28], meat cooked in a water bath at 100°C for 60 minutes had the lowest shear force value due to complete solubilization of collagen [37]. In comparison to other camel meat samples in this study, boiled samples displayed lower values of all textural attributes compared to other cooked samples. This is consistent with SDS-PAGE results (Figure 2) that show maximum protein degradation in boiled samples.

## CONCLUSION

Camel meat’s physicochemical properties, lipid and protein composition were altered by all cooking methods. The boiling method exhibited a lower degree of lipid oxidation and acceptable levels of cooking loss compared to other cooking methods. Boiling also resulted in greater protein degradation than other cooking methods, as shown by the lower hardness values of the boiled meat. Hence, boiling could be the more appropriate method for cooking camel meat since it can reduce lipid oxidation and toughness. On the other hand, microwaving presented the highest cooking loss and TBARS values. The results will aid consumers in determining the best cooking method for camel meat.

## Figures and Tables

**Table 1:** Effect of different cooking methods on proximate composition (%) and cooking loss (%) of the camel meat

Items	Fresh	Fried	Grilled	Microwaved	Boiled
Moisture	71.22±3.40^a^	54.23±3.10^d^	59.54±2.40^c^	62.37±2.80^c^	65.27±3.90^b^
Protein	22.58±2.20^d^	29.43±2.90^a^	27.44±3.10^b^	26.66±3.20^bc^	26.13±3.60^c^
Fat	5.25±0.67^d^	9.43±0.85^a^	7.22±1.32^b^	6.16±1.47^bc^	5.86±0.61^cd^
Ash	1.49±0.16^c^	2.28±0.25^a^	2.33±0.28^a^	2.21±0.14^a^	1.88±0.20^b^
Cooking loss	-	47.10±5.32^bd^	44.98±1.30^cb^	52.61±2.41^ad^	47.65±0.12^bd^

Values are mean±standard deviation (n = 3).

a–d Different superscripts within each row denote the significant differences among treatments (p<0.05).

**Table 2:** Effect of different cooking methods on texture profile of the camel meat

Samples	Hardness (g)	Cohesiveness	Springiness (mm)	Chewiness(mJ)
Fresh	1,133.33±69.60^e^	0.73±0.11^d^	6.20±0.90^c^	68.79±6.11^c^
Fried	2,327.30±101.03^b^	0.83±0.10^b^	8.25±0.92^a^	85.33±5.23^a^
Grilled	2,122.40±114.03^c^	0.77±0.05^c^	8.27±1.07^a^	76.85±6.98^b^
Microwaved	2,716.50±119.03^a^	0.89±0.12^a^	8.30±1.14^a^	84.13±5.18^a^
Boiled	1,923.50±83.03^d^	0.76±0.07^c^	7.80±0.94^b^	70.13±4.98^c^

Values are mean±standard deviation (n = 3).

a–d Different superscripts within the same column denote the significant differences among treatments (p<0.05).
